# Thermodynamic Analysis of Working Fluids for a New “Heat from Cold” Cycle

**DOI:** 10.3390/e22080808

**Published:** 2020-07-23

**Authors:** Ilya Girnik, Mikhail Tokarev, Yuri Aristov

**Affiliations:** Boreskov Institute of Catalysis, Ac. Lavrentiev av. 5, 630055 Novosibirsk, Russia; girnik@catalysis.ru (I.G.); tokarev@catalysis.ru (M.T.)

**Keywords:** adsorptive heating, methanol, ammonia, hydrofluorocarbons, water

## Abstract

Adsorptive Heat Transformation systems are at the interface between thermal and chemical engineering. Their study and development need a thorough thermodynamic analysis aimed at the smart choice of adsorbent-adsorptive pair and its fitting with a particular heat transformation cycle. This paper addresses such an analysis for a new “Heat from Cold” cycle proposed for amplification of the ambient heat in cold countries. A comparison of four working fluids is made in terms of the useful heat per cycle and the temperature lift. The useful heat increases in the row water > ammonia ≥ methanol > hydrofluorocarbon R32. A threshold mass of exchanged adsorbate, below which the useful heat equals zero, raises in the same sequence. The most promising adsorbents for this cycle are activated carbons Maxsorb III and SRD 1352/2. For all the adsorptives studied, a linear relationship *F* = *A*·Δ*T* is found between the Dubinin adsorption potential and the driving temperature difference Δ*T* between the two natural thermal baths. It allows the maximum temperature lift during the heat generation stage to be assessed. Thus, a larger Δ*T*-value promotes the removal of the more strongly bound adsorbate.

## 1. Introduction

Adsorptive Heat Transformation (AHT) is just at the interface between thermal and chemical engineering as the final goal is a heat conversion, whereas the specific means is a thermally driven adsorption process or chemical reaction [1]. This fast developing technology [2,3,4] aims to utilize renewable sources of heat that are deemed to replace fossil fuels soon. Low-temperature heat wasted from industrial, transport, and residential sources is another driving force for AHT cycles [5,6]. Advanced adsorption-based systems for cooling/heating are expected to compete with common vapour compression cycles that are no longer acceptable for ecological reasons. With zero ozone depletion and global warming potentials, adsorption chillers are environmentally benign. Their large-scale distribution requires a further study of AHT processes. It aims at accounting for both thermodynamic and kinetic issues, smart integration of components into a whole unit, improvement of adsorbent properties, etc. [2,4,7]. An in-depth thermodynamic study of AHT systems provides indispensable tools to outline the AHT limits and also assists in designing AHT units with better engineering properties [8,9].

The thermodynamic analysis is especially in demand when an AHT unit is driven by a low-temperature ambient heat, which is characterized by huge quantity and flux. However, its temperature is close to the temperature of the surroundings. Therefore, the quality of the heat is low and has to be properly evaluated. One such cycle has recently been proposed for upgrading the ambient heat during the wintertime in cold countries [10]. It was called “Heat from Cold” (HeCol) because a cold ambient is inherently necessary for its implementation and good performance.

**Figure 1:**
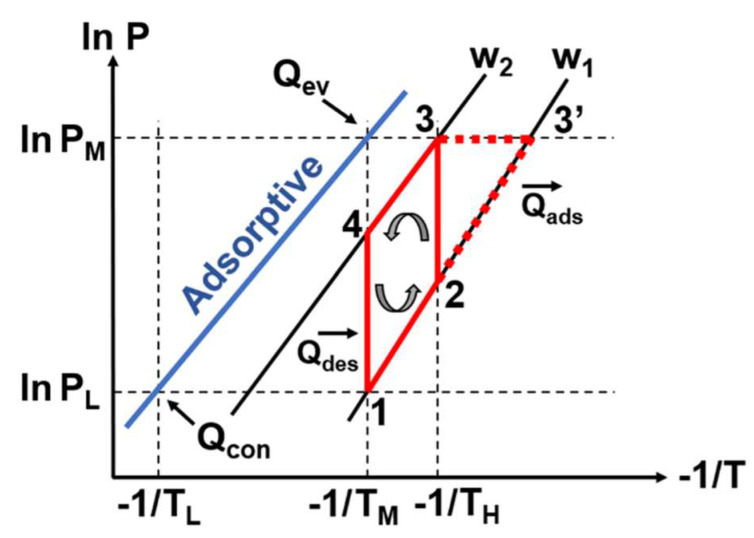
P-T diagram of isothermal (1-2-3-4) and non-isothermal (1-2-3′-3-4) HeCol cycles [12].

The isothermal HeCol cycle can be presented in a *P-T* diagram as two isotherms and two isosteres (Figure 1). Its main feature is the way of adsorbent regeneration: it occurs due to a drop in vapour pressure from *P*_4_ to *P*_1_ at a constant temperature of the adsorbent *T*_M_. The final pressure *P*_1_ is kept low because the condensation temperature equals the ambient temperature *T*_L_. In cold territories, the latter can be as low as (−50) − (−20) °C. The useful heat generation is caused by a pressure jump from *P*_2_ to *P*_3_ at a constant temperature of the adsorbent *T*_H_, sufficient for heating. Somewhat higher useful heat temperature *T*_3′_ can be obtained using the non-isothermal variant of HeCol (2-3′-3 in Figure 1). The evaporation and adsorption temperature *T*_M_ equals the temperature of another natural thermal bath, which is a reservoir of non-freezing water, such as a river, lake, sea, groundwater (see refs. [10,11] for more detail, including the HeCol basic thermodynamics).

Methanol was used as an adsorptive in the first theoretical [13] and experimental [11,14,15] studies of the HeCol cycle. These studies confirmed that the non-isothermal HeCol cycle could, in principle, be implemented, and the temperature level of generated useful heat can be suitable for heating (*T*_3′_ > 35 °C). The two main disadvantages revealed are (i) rather modest specific useful heat (300–400 J/g_adsorbent), and (ii) low vapour pressure of methanol that can limit the average specific heating power [11]. These drawbacks give a motivation for improving the HeCol cycles, for instance, by substituting methanol with adsorptive having better performance.

In this paper, water, ammonia, difluoromethane (R32), 1,1,1,2-tetrafluoroethane (R134) and 1,1-difluoroethane (R152) are considered as alternative working fluids. Water is widely used in common AHT units because of its high evaporation enthalpy and safety properties [4,5]. Ammonia is mainly used as a refrigerant for adsorptive deep freezing and ice making [8,16]. R32, R134, and R152 represent a family of hydrofluorocarbons that have insignificant ozone depletion potential and low global warming potential [17,18]. A brief overview of the thermodynamic properties of these alternative working fluids is given in Table 1.

The effect of the working fluid on the useful heat per cycle is studied in Section 2. As the useful heat generated in the HeCol cycle is essentially determined by the specific mass of adsorbate ∆*w = w*_2_ − *w*_1_ [g_adsorbate/g_adsorbent] exchanged in this cycle, a unified approach for evaluating this mass is described in Section 3 and Section 4. The Dubinin adsorption potential [19] is considered as a main thermodynamic parameter for this analysis because it can be used to describe the cycle boundary conditions and at the same time to characterize the adsorbent affinity to an adsorptive. This potential is also used in Section 5 to evaluate the maximal temperature lift during the heat rejection stage (2-3′ in Figure 1). Finally, a brief analysis is made to assess the effect of the vapour pressure on the diffusional flux under conditions of HeCol cycles (Section 6).

## 2. Effect of the Working Fluid on the Useful Heat per Cycle

In this Section, the question “How does the adsorptive nature affect the performance of a typical HeCol cycle?” is considered in terms of the specific useful heat *Q*_us_ [J/g_adsorbent] generated per cycle and related to the adsorbent mass *m*_a_. This heat is equal to the adsorption heat *Q*_ads_ released at stage 2-3 (or 2-3′) excluding the sensible heat *Q*_sen_ needed for isosteric heating 1-2:

(1) $$Q_{\mathrm{us}}=Q_{\mathrm{ads}}-Q_{\mathrm{sen}}=\Delta H_{\mathrm{ads}}\Delta w-CM\left(T_{H}-T_{M}\right)/m_{a},$$

where Δ*H*_ads_ is the specific adsorption heat [J/g_adsorbate], *C* and *M* are the overall specific heat capacity and the mass of inert components. The specific heat of working fluid in the vapour phase is neglected as its mass is small as compared with that in the adsorbed state. The values of *C* and *M* depend on the chosen control volume, as suggested in ref. [20]. In the considered case, the control volume includes adsorbent, adsorbate, and metal heat exchanger, which altogether can be called “adsorbent – heat exchanger” unit (AdHEx), so that Equation (1) can be re-written as

(2) $$\begin{array}{c} Q_{\mathrm{us}}=Q_{\mathrm{ads}}-\left(Q_{\mathrm{sen}}^{\mathrm{adsorbent}}+Q_{\mathrm{sen}}^{\mathrm{adsorbate}}+Q_{\mathrm{sen}}^{\mathrm{metal}}\right)=\Delta H_{\mathrm{ads}}\Delta w-[C_{\mathrm{adsorbent}}+C_{\mathrm{adsorbate}}(\Delta w/2\\ +w_{1})+C_{\mathrm{Al}}M_{\mathrm{Al}}/m_{a}]\left(T_{H}-T_{M}\right). \end{array}$$

where *w*_1_ is the initial adsorbate content. Equation (2) accounts for the sensible heat of adsorbate, which is an essential improvement as compared with the simplified approach of ref. [20].

For evaluating the effect of potential adsorptive, the useful heat *Q*_us_ is calculated for four working pairs with activated carbon (AC) as an adsorbent and various adsorptives (water, methanol, ammonia, and difluoromethane CH_2_F_2_, R32). For water, the AC is an ultramicroporius activated carbon fibre studied in [21]. For all other vapours, the AC is a commercial carbon SRD 1352/2, which was tested for AHT cycles with methanol [22], ammonia [16], and R32 [23]. R32 was selected for this analysis as it has zero ozone depletion potential, low GWP, and excellent heat transfer parameters. Its evaporation heat is relatively large as compared with the majority of common HFC refrigerants. 

A plate-tube finned heat exchanger (Yamaha Aerox) made of aluminium with the dimensions 190 × 200 × 30 mm^3^ is used as a reference HEx as it was tested for the HeCol prototype in refs. [14,15]. The mass of carbon loaded into the HEx *m*_a_ = 0.15 kg and the HEx mass *M*_Al_ = 0.50 kg. The specific heat capacity of each inert component is presented in Table 2. The adsorption heat Δ*H*_ads_ is calculated from equilibrium data of refs. [16,21,22,23] (Table 3). To be specific in calculating the sensible heat, the analysis is made for a typical HeCol cycle with *T*_L_ = −20 °C, *T*_M_ = 20 °C, and *T*_H_ = 40 °C [14].

**Figure 2:**
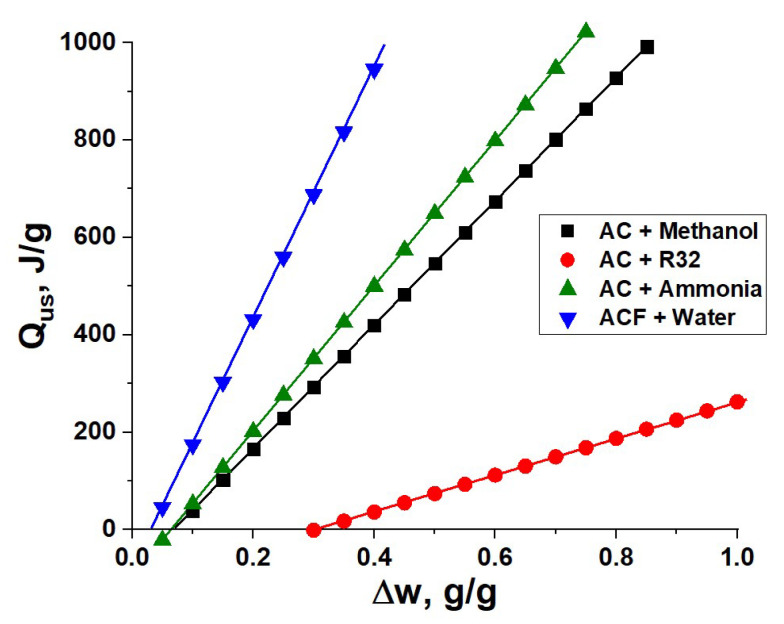
Useful heat as a function of the adsorbate mass exchanged in the HeCol cycle for the selected working pairs listed in the graph (see other details in the text).

Figure 2 shows that the useful heat linearly increases at larger adsorbate exchange ∆*w*. The following conclusions can be made:

1. water is the best working fluid,
2. there is a little difference between methanol and ammonia,
3. dramatically smaller useful heat can be obtained when R32 is used.

The main contribution to the useful heat comes from the adsorption heat Δ*H*_ads_ that determines the slope of the straight line in Figure 2. This heat is 6.5 and 3.2 times smaller for R32 as compared with water and methanol (Table 3). Since R32 has a relatively large for HFCs evaporation heat, its adsorption heat is also larger than for the majority of commercial HFCs. From the thermodynamic point of view, any HFC can hardly be used in HeCol cycles, unless the exchanged mass ∆*w* is extraordinarily large to compensate for the low adsorption heat. For instance, the uptake ∆*w*′ needed to obtain a desirable *Q*_us_-value of 500 J/g is common for water (0.23 g/g), advanced for methanol and ammonia (ca. 0.4–0.45 g/g), and poorly realistic for R32 (1.63 g/g) (Table 3). The adsorption heat also affects a threshold exchange ∆w* = [*C*∙*M*∙(*T*_H_ − *T*_M_)]/(Δ*H*_ads_∙*m*_a_), at which the released adsorption heat in Equation (1) is exactly equal to the sensible heat of AdHEx unit. If ∆*w* < ∆w*, the useful effect is zero (Figure 2). The threshold value is much larger for R32 than for other adsorptives (Table 3).

## 3. General Description of the HeCol Cycles in Terms of the Dubinin Adsorption Potential

**Figure 3:**
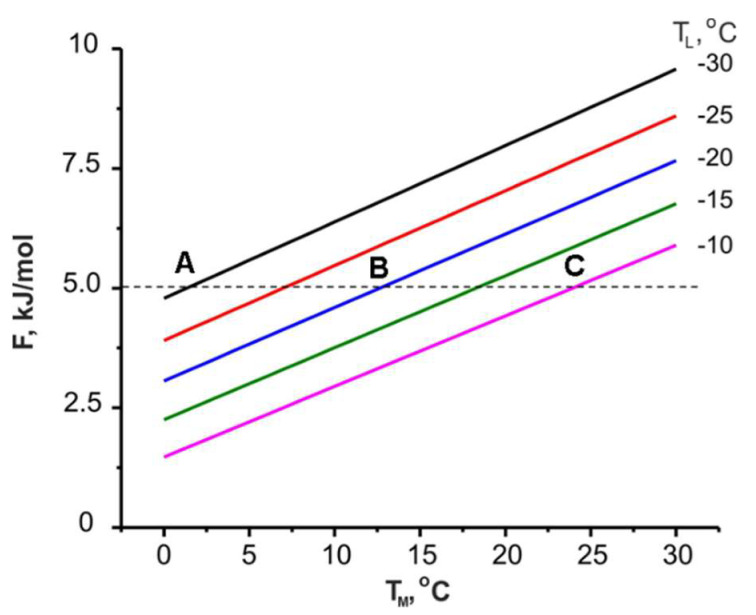
Adsorption potential *F*_1-2_ corresponding to the weak adsorption isostere as a function of the methanol desorption temperature *T*_M_ at various temperatures *T*_L_ of the condenser (ambient air).

Figure 2 illustrates the functional dependence of the useful heat on the adsorbate mass ∆*w* exchanged in the cycle, which has to be maximized. It means that an optimal adsorbent should ensure, on the one hand, effective adsorption of vapour at heat generation stage (2-3 or 2-3′), and on the other hand, it’s easy giving off at desorption stage (4-1) of the HeCol cycle. To formulate a quantitative criterion for the adsorbent desirable for the adsorptives involved, the Polanyi invariance principle was used [28]. It postulates a one-to-one correspondence between the volume of adsorbed vapour and the Dubinin adsorption potential *F* [19]. The validity of this principle was justified in [29] for many working pairs promising for AHT. For these pairs, it was shown that the use of this potential for analyzing AHT cycles is very convenient because the cycle borders depend on only one parameter (*F*) instead of the common two (*P* and *T*). On the other hand, this potential can be considered as a quantitative measure of the affinity between the adsorbent and the adsorptive [30]. For instance, if an adsorbent (or particular adsorption center) has the affinity to methanol vapour, let say, *F* = 5.0 kJ/mol, and the methanol vapour pressure corresponds to the condenser (ambient) temperature *T*_L_ = −30 °C, methanol can be desorbed at the desorption temperature *T*_M_ ≥ 1 °C (point A in Figure 3). Thus, any non-freezing water basin can be used as a heat source for desorption. At a higher ambient temperature of −20 and −10 °C, the minimal desorption temperature increases to 13 and 24 °C (points B and C in Figure 3). At stronger affinity, the methanol desorption occurs at large *F*_1-2_-value. Accordingly, desorption requires high *T*_M_ and low *T*_L_. Therefore, a trade-off between these parameters has to be reached for the adsorbent to be optimal.

The values *F*_1-2_ and *F*_3-4_ corresponding to the weak *w*_1_ and rich *w*_2_ isosteres are calculated for all adsorptives considered here at various boundary temperatures *T*_L_, *T*_M_, and *T*_H_ of the HeCol cycle and collected in Table 4 and Table 5.

The main finding following from Table 4 and Table 5 is that the properties of adsorbent optimal for HeCol cycles vary greatly depending on climatic conditions (*T*_L_, *T*_M_) and the temperature level *T*_H_ of heat needed for a Consumer. For instance, if the temperature difference Δ*T* = (*T*_M_ − *T*_L_) is small, the optimal adsorbent should bind adsorptive weaker and give it off easier, means, at a lower *F*-value. *Vice versa*, at the adsorption stage, this adsorbent ensures a small temperature lift Δ*T* = (*T*_3′_ − *T*_2_) due to its low affinity to adsorptive (see Section 5). These tables also permit the evaluation of the optimal affinity to adsorptive for adsorbent to be used in a cycle with given temperatures of regeneration *T*_M_ and environment *T*_L_.

**Figure 4:**
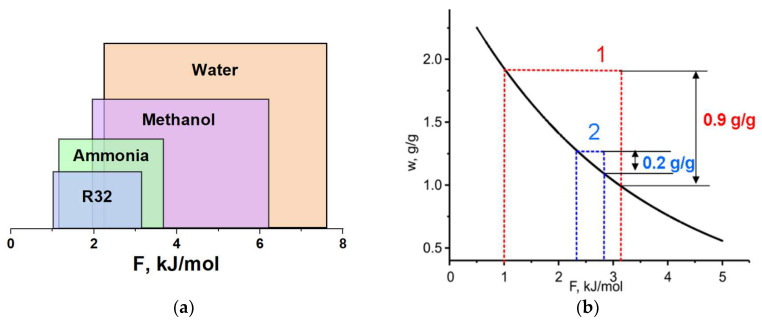
(**a**)—Cycle windows for various adsorptives at the fixed boundary temperatures *T*_L_/*T*_M_/*T*_H_ = −20/20/35 °C. (**b**)—Universal adsorption isotherm of R32 on the phenolic rubber carbon together with the windows for the “mild” (1) and “harsh” HeCol cycles (2) (see text for more details).

Figure 4a shows the cycle windows for various adsorptives at the cycle boundary temperatures *T*_L_/*T*_M_/*T*_H_ = −20/20/35 °C. The boundary *F*-values of this particular HeCol cycle much differ for the adsorptives analyzed. The adsorption isobar of the optimal adsorbent should increase sharply in the *F*-range between *F*_1-2_ and *F*_3-4_. Appropriate adsorbents should be selected from the literature or intently synthesized. Several examples of such a selection are given in the next section.

## 4. Evaluation of the Adsorbate Amount Exchanged in the HeCol Cycle

### 4.1. Adsorbents of Methanol

This evaluation is, first, made for methanol as a reference adsorptive. Equilibrium data on the methanol vapour adsorption are taken from the literature [31,32] for several commercial ACs promising for AHT. The data are analyzed based on the cycle window approach for two HeCol cycles. As the first cycle with *T*_L_/*T*_M_/*T*_H_ = −20/20/35 °C is less difficult for realization, it is called as “mild”. The other cycle with *T*_L_/*T*_M_/*T*_H_ = −30/3/35 °C is much more difficult (“harsh” cycle). The main findings of the analysis are summarized in Table 6.

Activated carbon Maxsorb III (Kensi Coke and Chemicals Co. Ltd., Amagasaki, Japan) has a maximum methanol adsorption capacity for both analyzed HeCol cycles. In the “mild” cycle, this carbon exchanges as much as Δ*w* = 0.82 g/g. This enormous uptake corresponds to the specific useful heat ca. 950 J/g as can be roughly estimated from Figure 2. For this cycle, quite large methanol exchange (0.35 g/g) is found for SRD1352/2 tested in Section 2 (*Q*_us_ = 360 J/g). For other carbons with the large specific surface area, the Δ*w*–value is promising for the “mild” HeCol cycle (0.17–0.44 g/g). At lower ambient temperature *T*_L_ = −30 °C, Δ*w* increases to 0.26–0.47 g/g. Under conditions of the “harsh” cycle, the exchanged mass decreases significantly for all studied carbons (Table 6); MaxSorb III remains the best one (Δ*w* = 0.17 g/g). With a decrease in *T*_L_ to −40 °C, this mass increases to 0.34 g/g. Thus, a cold ambient can help in obtaining larger adsorptive exchange Δ*w* and, hence, greater useful heat (Figure 2). The methanol exchange of other tested carbons in the latter cycle also increases to 0.15–0.21 g/g.

### 4.2. Adsorbents of Ammonia

A similar analysis is made for carbonaceous adsorbents of ammonia. The most comprehensive set of data on the ammonia adsorption equilibrium on many commercial carbons was reported in [16] to assess the carbon applicability for AHT. The data are presented as coefficients *w*_o_, *k*, and *n* in the Dubinin-Radushkevich equation presented as $w=w_{o}\cdot exp\left[-k{\left(\frac{T}{T_{\mathrm{sat}}}-1\right)}^{n}\right]$, where *w*_o_ is the ammonia uptake under saturation conditions, *T* is the carbon temperature, *T*_sat_ is the saturation temperature corresponding to the vapour pressure. These coefficients were used for calculating the ammonia uptake corresponding to weak *w*_1_ and rich *w*_2_ isosteres, as well as the ammonia mass Δ*w* exchanged in the reference HeCol cycles (Table 7).

For the “mild” cycle, only a few carbons (SRD1352/2, ACF-20, C-2132, and MSC-30) exchange practically interesting mass of ammonia per cycle Δ*w* = 0.23–0.34 g/g (Table 7). The specific useful heat estimated from Figure 2 ranges between 250 and 410 J/g. The apparent density of these last carbons is quite low (0.10–0.26 g/cm^3^), which results in the modest ammonia exchange related to a unit volume (0.05–0.10 g/cm^3^). However, the density can be increased by a factor of 1.5–3 by compaction of the initial carbon (powder, fibre, etc.) in a denser structure [16]. 

**Figure 5:**
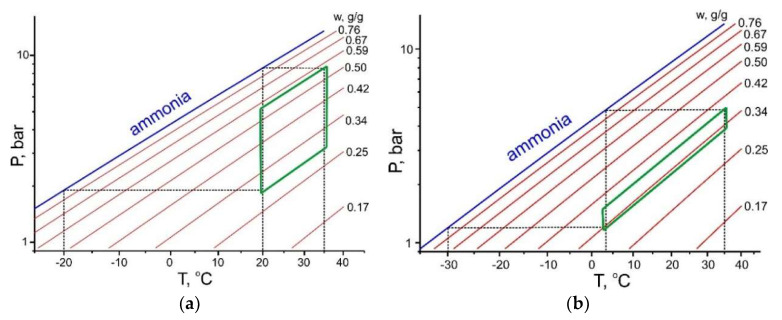
HeCol cycles plotted for the working pair “ammonia - carbon SRD1352/2” at two boundary conditions *T*_L_/*T*_M_/*T*_H_ = −20/20/35 °C (**a**) and −30/3/35 °C (**b**).

For the “harsh” cycle, the maximum ammonia exchange is 0.05 g/g that is not sufficient even to compensate the sensible heating of the AdHEx unit (compare with the threshold exchange ∆w* in Figure 2 and Table 3). Therefore, none of the carbons studied can be used in this cycle, and new ammonia adsorbents that are better suited to the cycle are required. The graphical illustration displayed in Figure 5 shows a difference between the “mild” and “harsh” HeCol cycles plotted for the working pair “ammonia - carbon SRD1352/2”. The latter cycle is very “narrow” so that the mass of ammonia exchanged is small (Δ*w* = 0.05 g/g).

In general, commercial carbons are characterized by sufficiently strong affinity to ammonia, so that the large driving force (*T*_M_ − *T*_L_) > 20–30 °C is commonly required for regeneration. The best carbons allow a useful heat of 250–410 J/g to be obtained per the “mild” HeCol cycle. According to this indicator, these carbons can compete with the methanol adsorbents (Section 4.1). Besides, the pressure of ammonia at both adsorption and desorption stages (1 bar and more) is much higher than that of methanol (see Section 6). It can be profitable for enhancing the rate of ad/desorption and the specific useful power of HeCol unit.

### 4.3. Adsorbents of Water

From the thermodynamic point of view, water is the best working fluid for including the HeCol cycle (Figure 2). However, water has the following practical disadvantages:

1. high melting temperature of 0 °C. Ice formation in the AHT evaporator/condenser at the ambient temperature below 0 °C limits the application of water as adsorptive;
2. low equilibrium vapour pressure. It can dramatically reduce mass transfer and adsorption rate.

It is very promising to use water vapour as adsorptive in the HeCol cycle, but special measures should be made to avoid ice formation in the condenser. Two ways have been proposed and tested in the literature: (i) to mix pure water with ethylene glycol as an anti-freezing agent [33], and (ii) to use an aqueous salt solution instead of pure water [12,34]. One more way to reduce the melting temperature could be confinement of water [35] or an aqueous salt solution [36] into a host matrix with tiny pores. The latter approach has not been analyzed yet and needs a special study. 

The comparative analysis was performed in [12] for the typical HeCol cycle with (*T*_L_/*T*_M_/*T*_H_ = −20/20/40 °C with methanol and a eutectic aqueous solution of CaCl_2_ as a liquid in the condenser. The composites (21 wt.%)LiCl/(silica gel) and (33 wt.%)CaCl_2_/(silica gel) were used as sorbents of both water and methanol vapour. This study revealed that:

1. the salt addition results in lower vapour pressure over the salt solution as compared with pure water/ice. The cycle boundary pressures and the uptake variation reduce appropriately. Despite this decrease, the specific useful heat remains much larger than that for methanol as adsorptive: 870 J/g versus 520 J/g;
2. the water adsorption dynamics is quite fast and ensure the initial useful power Win = 11.3 kW/kg. The driving force for desorption is smaller, and this process is slower (Win = 2.1–3.4 kW/kg). A smart trade-off between the HeCol useful heat and specific power has to be reached to make both output parameters acceptable in practice.

Thus, the substitution of methanol with water as adsorptive can help to increase the useful heat; however, at the expense of lower heating power as the water vapour pressure is lower than for methanol. More details on applying water as adsorptive in typical HeCol cycles can be found elsewhere [12].

### 4.4. Adsorbents of Hydrofluorocarbons (HFCs)

For HFCs, fewer equilibrium data are available in the literature than for the other three adsorptives considered, which can be due to its low evaporation heat. On the other hand, the HFC high vapour pressure at typical temperatures of the HeCol cycle (Table 8) can be profitable for increasing the adsorption rate (Section 6). Adsorption of HFCs on commercial carbons was studied intensively by Saha with co-authors (see, e.g., [37,38,39,40]. Three ozone-friendly HFCs (R32, R134, *1,1,1,2-tetrafluoroethane*, and R152, *1,1-difluoroethane*) can be identified as the most perspective because of their relatively large uptake exchange in the “mild” and “harsh” HeCol cycles (Table 9).

**Figure 6:**
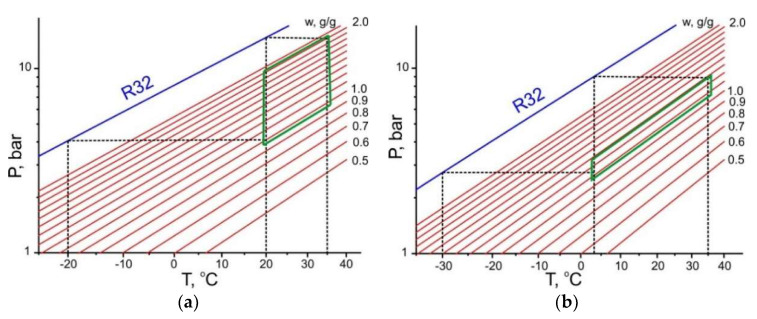
HeCol cycles plotted for the working pair “R32—phenolic rubber carbon” for the “mild” (**a**) and “harsh” (**b**) HeCol cycles.

From the literature data, the maximum adsorption uptake of R32 (up to 2.3 g/g) is found for the innovative carbon synthesized from phenolic rubber [38]. As mentioned in Section 3, the equilibrium adsorption uptake on this carbon is a unique function of the Dubinin adsorption potential (Figure 4b). For this working pair, a quite large difference between the “mild” and “harsh” cycles, plotted from the equilibrium data of ref. [38], is illustrated in Figure 6. The appropriate uptake difference is 0.90 and 0.20 g/g, respectively (Table 9 and Figure 4b). However, even for this outstanding adsorbent, the useful heat for the “mild” cycle estimated from Figure 2 is rather modest (ca. 220 J/g), so that it is not competitive with other working fluids tested in this paper. For the “harsh” cycle, the equilibrium exchange is smaller than the threshold exchange for R32 (∆w* = 0.20–0.25 g/g, Figure 2) for all tested pairs.

Even lower values of the exchanged uptake are found for R134 and R152 (Table 9). Only for the innovative carbon Maxsorb III it is decently larger than the threshold exchange. The useful heat still does not exceed 100 J/g. Hence, they all moreover cannot be used in any HeCol cycle. Another drawback comes from the low density of the carbons already mentioned in Section 4.2. Due to this, the specific useful heat related to the carbon unit volume (J/cm^3^) appears to reduce by a factor of 2–10.

## 5. Link between the Adsorbent Affinity and the Driving Temperature Differences

Section 3 indicates that the adsorbent affinity to the adsorptive and the temperature difference between two natural thermostats Δ*T* = (*T*_M_ − *T*_L_) are linked and should be in harmony for effective implementation of HeCol cycle. This temperature difference can be considered as a thermodynamic driving force for vapour desorption. The adsorption potential *F* is assumed to be a quantitative measure of the adsorbent affinity. For methanol removal at stage 4-1, there is a linear relationship between these values

**Figure 7:**
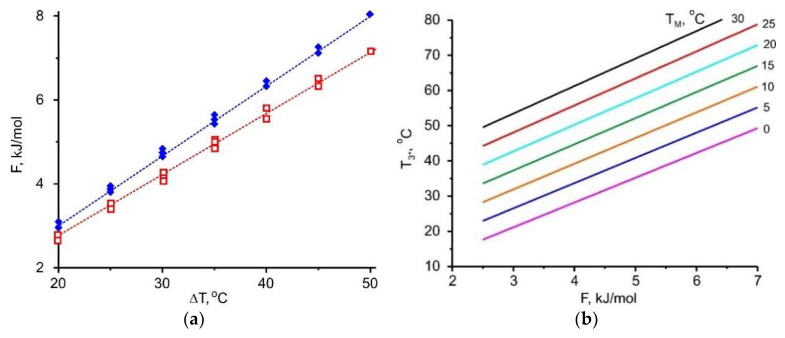
(**a**)—Relationship between the Dubinin adsorption potential *F* for methanol and the driving temperature difference Δ*T* = (*T*_M_ − *T*_L_) (∧) or (*T*_3*_ − *T*_M_) (∀); (**b**)—dependence of the maximum heating temperature *T*_3*_ at the adsorption stage on the Dubinin adsorption potential *F* at various *T*_M_.

(3) F(ΔT)=A·ΔT

with a slope *A* of (162 ± 10) J/(mol K) (the upper line in Figure 7a). Thus, a larger temperature difference between a non-freezing water basin and the ambient air Δ*T* = (*T*_M_ − *T*_L_) promotes the removal of more strongly bound adsorbate, which corresponds to higher *F*-value: the cold ambient facilitates the desorption and adsorbent regeneration.

Let us estimate what affinity for methanol should have an adsorbent, if for its regeneration, heat with *T*_M_ = 5 °C and “cold” with *T*_L_ = −20 °C (Δ*T* = 25 °C) are available: *F* = 3.70 kJ/mol. If the adsorbent has a weaker affinity, it can be regenerated easier, means, at lower *T*_M_ or higher *T*_L_. If it has a stronger affinity, higher *T*_M_ or lower *T*_L_ are needed for its regeneration. 

Together with the useful heat, another important characteristic of the HeCol process is a maximum temperature lift Δ*T* = (*T*_3*_ − *T*_M_) achievable during the heat generation process (stage (1-3*) for the non-isothermal cycle (Figure 1). It determines the maximum rise in the temperature of the natural water basin in the particular HeCol cycle and permits to assess whether the generated heat can be used for heating purposes. For evaluating this temperature lift, the function *F*(Δ*T*) is plotted for methanol adsorption (the lower line in Figure 7a); it is a straight line with *A* = (134 ± 10) J/(mol K). If there is no adsorption-desorption hysteresis, the methanol adsorption proceeds at the same Dubinin potential *F* = 3.70 kJ/mol at which desorption did. For such the adsorbent, the maximum lift Δ*T* equals 28 °C. This lift is larger than the driving temperature difference Δ*T* = (*T*_M_ − *T*_L_) = 5 °C − (−20 °C) = 25 °C at the desorption stage. The temperature *T*_max_ = *T*_M_ + Δ*T* = 5 °C + 28 °C = 33 °C can be obtained during the heat generation stage. It can be of practical interest (e.g., for floor heating systems). The lift increases for adsorbents with stronger affinity to methanol, so that a more valuable heat can be obtained. 

**Figure 8:**
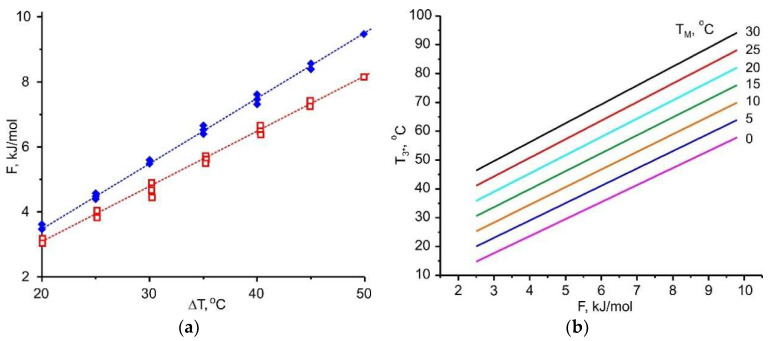
(**a**)—Relationship between the Dubinin adsorption potential *F* for water and the driving temperature difference Δ*T* = (*T*_M_ − *T*_L_) (∧) or (*T*_3*_ − *T*_M_) (∀); (**b**)—dependence of the maximum heating temperature *T*_3*_ at the adsorption stage on the Dubinin adsorption potential *F* at various *T*_M_.

**Figure 9:**
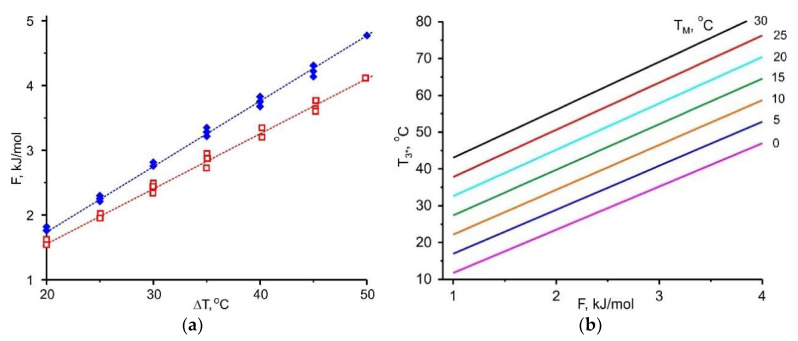
(**a**)—Relationship between the Dubinin adsorption potential *F* for ammonia and the driving temperature difference Δ*T* = (*T*_M_ − *T*_L_) (∧) or (*T*_3*_ −*T*_M_) (∀); (**b**)—dependence of the maximum heating temperature *T*_3*_ at the adsorption stage on the Dubinin adsorption potential *F* at various *T*_M_.

**Figure 10:**
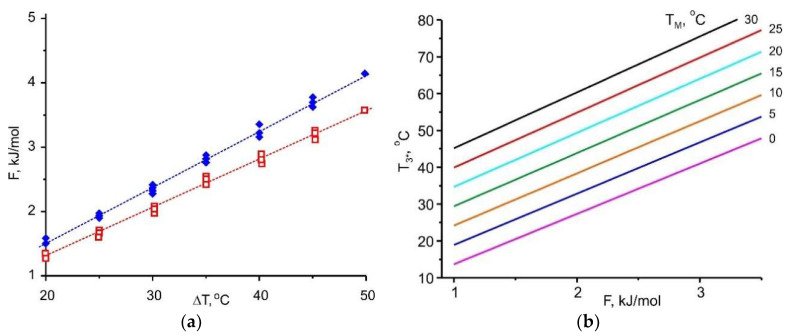
(**a**)—Relationship between the Dubinin adsorption potential *F* for hydrofluorocarbon R32 and the driving temperature difference Δ*T* = (*T*_M_ − *T*_L_) (∧) or (*T*_3*_ − *T*_M_) (∀); (**b**)—dependence of the maximum heating temperature *T*_3*_ at the adsorption stage on the Dubinin adsorption potential *F* at various *T*_M_.

Linear relationship (3) is found for all the adsorptives involved (Figure 7, Figure 8, Figure 9 and Figure 10); the appropriate slopes *A* are displayed in Table 10. The inverse value 1/*A* = 14.1, 12.0, 7.5, and 5.9 K/(kJ/mol) for R32, ammonia, methanol, and water, respectively, allows the maximum temperature lift to be estimated for any adsorbent with a given affinity *F*.

Figure 7, Figure 8, Figure 9 and Figure 10 demonstrate the common feature for all the adsorptives studied; namely, a linear dependence ∆*F* = *A*·Δ*T*, where ∆*T* = (*T*_M_ − *T*_L_) for desorption and (*T*_3*_ − *T*_M_) for adsorption. It gives a quantitative link between the adsorbent affinity to the given adsorptive and the temperature driving difference. This relation is very useful as it helps in choosing adsorbents optimal for the given conditions of HeCol cycle:

1. for various ambient conditions, means, at different temperature sets (*T*_L_, *T*_M_), the adsorbents required for the implementation of HeCol cycle can vary greatly: At a small driving force ∆*T* = (*T*_M_ − *T*_L_), the adsorbent should give off an adsorbate at a lower ∆*F*-value. It corresponds to a lower affinity between the adsorbent and the adsorbate. Thus, Equation (3) can be the base for establishing a conditional scale of adsorbent strength;
2. the difference Δ*T* = (*T*_3*_ − *T*_M_) shows how much the temperature level *T*_M_ of the non-freezing water source (lake, river, sea, underground water, etc.) can be amplified in the HeCol cycle using the adsorbent with a given affinity. The temperature *T*_3*_ = *T*_M_ + Δ*T* defines the maximum heating level can be obtained during the adsorption (heat generation) stage; hence, possible applications of this heat.

This thermodynamic analysis shows that a larger temperature difference (*T*_M_ − *T*_L_) of natural heat baths helps to remove a more firmly bound adsorbate, which corresponds to higher *F*-value. Interesting that at the adsorption stage it is possible to obtain a useful temperature difference (*T*_3*_ − *T*_M_) that is somewhat larger than the temperature difference (*T*_M_ − *T*_L_) applied to drive the desorption stage (Figure 7a, Figure 8a, Figure 9a and Figure 10a). By using a “stronger” adsorbent at the adsorption stage, a higher temperature can be obtained, i.e., more valuable heat for heating.

## 6. Brief Dynamic Considerations

The main aim of this study is the thermodynamic analysis of various working fluids for the HeCol cycle. The adsorption and, especially, desorption dynamics is also significant for the efficient cycle operation as both processes occur at low pressure and temperature. In this section, the effect of the gas (vapour) pressure of adsorption dynamics is briefly considered. A more detailed dynamic analysis should be the subject of a separate study. 

The main motivation to consider ammonia and HFCs as adsorptives instead of methanol is their higher saturated vapour pressure – bars instead of tens of millibars (Table 1). Such a substitution results in the proper rise in the boundary pressures of HeCol cycles (Table 11) occurring at low temperatures (−40 °C < *T* < 40 °C). These severe conditions can slow down vapour transport dramatically and, hence, reduce the adsorption rate and the cycle specific power. In this section, the effect of the gas (vapour) pressure of adsorption dynamics is briefly considered.

To highlight the effect of vapour pressure, a case when the total adsorption rate is limited by vapour transport inside an adsorbent grain or layer is considered, so that the diffusional flux *A*_dif_ is

(4) Adif=SDdPdr

where *S* is the mass transfer surface area, *D* is the vapour diffusivity, and d*P*/d*r* is the vapour pressure gradient in the adsorbent grain/layer. Both vapour diffusivity and pressure gradient rise at higher vapour pressure *P*. In a straight cylindrical pore, the pressure dependence of gas diffusivity is determined by the transport mechanisms: the Knudsen diffusivity *D*_Kn_ does not depend on pressure, the molecular (*D*_mol_ ~ 1/*P*) and Poiseuille (*D*_Pois_ ~ *P*^2^) ones are dependent. The overall diffusivity in pores *D*_p_ can be evaluated as [45]

(5) $$D_{p}\approx D_{\mathrm{Pois}}+\frac{D_{\mathrm{Kn}}D_{\mathrm{mol}}}{D_{\mathrm{Kn}}+D_{\mathrm{mol}}}$$

**Figure 11:**
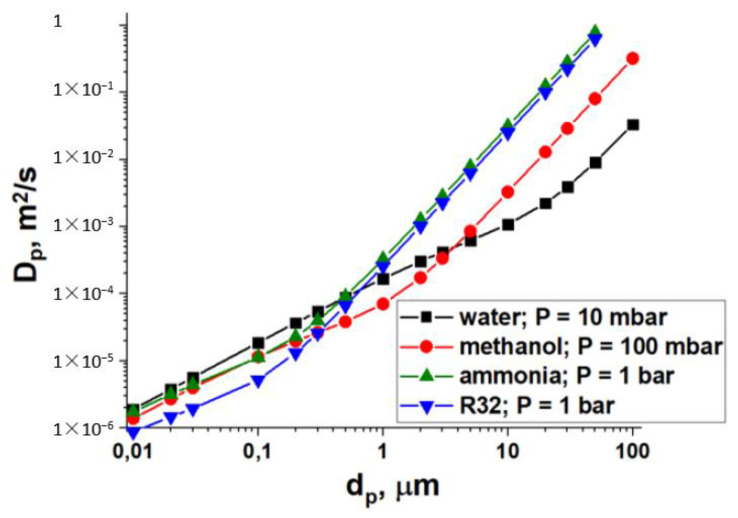
Overall diffusivity *D*_p_ in a straight cylindrical pore as a function of the pore diameter *d*_p_ for water (■), methanol (●), ammonia (▲), and R32 (▼) at *T* = 0 °C at various vapour pressures *P* listed in the graph. Calculated from Equation (5).

The overall diffusivity in pores *D*_p_ calculated at vapour pressures 0.01, 0.1, 1.0, and 1.0 bar for water, methanol, ammonia, and R32, respectively, is displayed in Figure 11. These pressures are typical for HeCol cycles, utilizing these adsorptives (Table 11). The most favourable viscous flow regime is established in pores larger than ca. 30 μm, 3 μm, 0.3, and 0.2 μm for water, methanol, ammonia, and R32, respectively. Under this mode, the *D*_p_-values for ammonia and R32 are almost equal and significantly larger than for water and methanol. In smaller pores, a transient regime and then the Knudsen one come (Figure 11). The latter mode dominates when collisions of vapour molecules with the pore wall are more frequent than those between the molecules.

Thus, the use of ammonia and R32 can significantly increase the diffusional flux, first of all, in large (transport) pores due to the dominant contribution of the Poiseuille flux. In smaller pores (*d*_p_ < 1 μm), the diffusivities for these adsorptives get closer and are somewhat larger for lighter molecules, and the flux increases mainly due to larger vapour pressure gradient in the adsorbent grain/layer d*P*/d*r*.

## 7. Summary

A new “Heat from Cold” (HeCol) cycle has recently been proposed for amplification of the ambient heat in cold countries. Methanol was used as an adsorptive in the previous studies of the HeCol cycle and prototypes. These studies demonstrated the feasibility of HeCol cycles; however, a rather modest specific useful heat (300–400 J/g_adsorbent) and average specific heating power (300–400 W/kg_adsorbent) were obtained. Thus, the improvement of the HeCol cycle by substituting methanol with alternative adsorptives is an interesting topic. This paper aims at the thermodynamic study of the effect of adsorptive on the HeCol performance.

A comparison of four working fluids (methanol, water, ammonia, and R32) is made in terms of the useful heat generated per cycle and the maximum temperature lift at the heat generation stage. Commercial activated carbons are considered as adsorbents. The useful heat *Q*_us_ is found to increase in the row water > ammonia ≥ methanol > R32. A threshold adsorbate mass exchanged in the HeCol cycle ∆w* is found; below this mass, the useful heat equals zero. The ∆w*-value reduces in the same sequence as the useful heat increases. This study revealed that from the thermodynamic point of view, (i) water is the best working fluid; (ii) there is a little difference between methanol and ammonia; and (iii) much smaller useful heat can be obtained for R32.

The *Q*_us_-value is essentially determined by the mass of adsorbate ∆*w* exchanged in the cycle. The Dubinin adsorption potential *F* is used to plot the HeCol cycle windows and evaluate the exchanged mass for commercial carbon selected from the literature. Quite large uptakes of methanol, ammonia and R32 (up to 0.8–0.9 g/g) are found for advanced microporous carbons, like Maxsorb III and SRD 1352/2. Appropriate working pairs can be recommended for the HeCol implementation. Applicability of water as adsorptive is considered in more detail in [34].

The Dubinin potential *F* is also useful in evaluating the maximal temperature lift Δ*T* = (*T*_3*_ − *T*_M_) achievable at the heat rejection stage (2-3′ in Figure 1). This lift is another important characteristic of the HeCol process. In this case, the *F*-value serves as a quantitative measure of the adsorbent affinity to adsorptive. For all the adsorptives studied, a linear relationship *F* = *A*·Δ*T* is found, and the slopes *A* are tabulated. Hence, by using a “stronger” adsorbent at the adsorption stage, a higher temperature can be obtained, i.e., more valuable heat for heating.

To analyse the effect of adsorptive from a single perspective, various commercial carbons are considered in this paper as potential adsorbents of methanol, water, ammonia, and HFCs for HeCol cycles. This analysis can be extended to other commercial and innovative sorbents; first of all, to composites “salt in porous matrix” (CSPMs). Various CSPMs are widely tested for AHT and other important applications [46]. In particular, two composites (CaClBr/SiO_2_ and LiCl/SiO_2_) were tested in the HeCol prototypes [11,47,48]. The CSPMs possess an enhanced sorption capacity with respect to water, methanol, and ammonia. No CSPMs have been developed so far for sorbing hydrofluorocarbons. As a confined salt provides additional sorption, one can expect larger specific useful heat, when CSPMs are used instead of carbons [11]. However, this stronger bonding can be unfavourable for desorption dynamics.

## 8. Conclusions

The use of various working fluids, such as water, methanol, ammonia, and hydrofluorocarbon R32, in the new “Heat from Cold” cycle is analyzed from the thermodynamic point of view. Commercial activated carbons are considered as adsorbents. This study revealed that (i) water is the best working fluid; (ii) there is a little difference between methanol and ammonia; and (iii) much smaller useful heat can be obtained for R32. The specific useful heat generated per cycle increases at a larger mass of adsorbate exchanged. The most promising adsorbents for this cycle are activated carbons Maxsorb III and SRD 1352/2.

For all the adsorptives studied, a linear relationship between Dubinin adsorption potential *F* and cycle temperature lift Δ*T* is found. For obtaining more valuable heat (with a higher temperature level), the adsorbent with a high affinity to the adsorbate should be used. For such a “stronger” adsorbent, a higher temperature of the useful heat can be obtained; however, a larger temperature difference (*T*_M_ − *T*_L_) is needed for its regeneration.

## Figures and Tables

**Table 1:** Thermodynamic parameters of various working fluids.

Working Fluid	*T*_m_, °C	Δ*H*_e_^1^, kJ/mol (kJ/g)	*P* (0 °C), mbar	*P* (−10 °C), mbar	*P* (−20 °C), mbar
Water (pure)	0	44.2 (2.45)	6.1	2.6	1.03
Methanol	−97.6	37.5 (1.17)	39.8	19.9	9.6
Ammonia	−77.3	23.3 (1.37)	4180	2850	1880
R32	−136.0	14.6 (0.28)	8130	5825	4060
R134	−103.3	18.5 (0.18)	2930	2007	1335
R152	−117.0	19.0 (0.29)	2670	1850	1250

^1^ at 20 °C.

**Table 2:** Specific heat capacity *C* of inert components of the reference AdHEx.

Component	*C*, J/(kg K)	Ref.
Aluminium	903	[24]
Methanol	2490	[24]
Water	4180	[13]
Ammonia	2175	[25]
R32	2000	[26]
Activated carbon	820	[27]

**Table 3:** Adsorption heat Δ*H*_ads_, threshold exchange ∆w* and exchange ∆*w*’ needed to obtain *Q*_us_ = 500 J/g for the working pairs analyzed.

Working Pair	Δ*H*_ads_, kJ/g	Δw*, g/g	Δ*w′*, g/g
ACF + water	2.61	0.032	0.23
SRD1352/2 + methanol	1.30	0.069	0.46
SRD1352/2 + ammonia	1.52	0.064	0.40
SRD1352/2 + R32	0.40	0.303	1.63

**Table 4:** Adsorption potential *F*_1-2_ (kJ/mol) corresponding to isostere (1-2).

Adsorptive	Methanol			Ammonia			Water			R32		
*T*_M_, °C *T*_L_, °C	3	10	20	3	10	20	3	10	20	3	10	20
−20	3.62	4.68	6.21	2.12	2.76	3.67	4.57	5.82	7.60	2.08	2.36	3.14
−30	5.42	6.54	8.14	3.19	3.85	4.80	6.86	8.17	10.03	2.95	3.29	4.10
−40	7.40	8.52	10.20	4.36	5.05	6.04	9.36	10.73	12.68	3.98	4.31	5.16

**Table 5:** Adsorption potential *F*_3-4_ (kJ/mol) corresponding to isostere (3-4).

Adsorptive	Methanol			Ammonia			Water			R32		
*T*_M_, °C *T*_H_, °C	3	10	20	3	10	20	3	10	20	3	10	20
35	4.47	3.40	1.96	2.65	2.01	1.16	5.13	3.90	2.25	2.00	1.75	1.02
50	6.53	5.41	3.91	3.88	3.21	2.32	7.49	6.20	4.47	3.06	2.80	2.03

**Table 6:** Specific surface area *S*_sp_ of the selected porous carbons and mass of methanol Δ*w* (g/g) exchanged in the HeCol cycles analyzed.

Carbonaceous Adsorbent	*S*_sp_, m^2^/g	Δ*w*, g/g	
		*T*_L_/*T*_M_/*T*_H_ = −20/20/35 °C	*T*_L_/*T*_M_/*T*_H_ = −30/3/35 °C
Maxsorb III	3150	0.82	0.16
CarboTech C40/1	1290	0.36	0.089
SRD1352/2	1630	0.35	0.095
ACM-35.4	1200	0.23	0.05

**Table 7:** Ammonia uptakes *w*_1_ and *w*_2_ (Figure 1) and specific mass of ammonia Δ*w* exchanged in the HeCol cycles analyzed.

Cycles Analysed		*T*_L_/*T*_M_/*T*_H_ = −20/20/35 °C			*T*_L_/*T*_M_/*T*_H_ = −30/3/35 °C		
Carbonaceous Adsorbent	*w*_o_	*w*_1_	*w*_2_	Δ*w, g/g* (g/cm^3^)	*w*_1_	*w*_2_	Δ*w, g/g* (g/cm^3^)
LM001	0.27	0.17	0.24	0.07 (0.05)	0.18	0.19	0.01 (0.007)
LM127	0.36	0.19	0.29	0.10 (0.075)	0.21	0.225	0.015(0.011)
LM128	0.33	0.18	0.27	0.09 (0.065)	0.20	0.215	0.015(0.011)
LM279	0.38	0.20	0.31	0.11 (0.09)	0.21	0.235	0.02 (0.017)
KOH-AC	0.625	0.32	0.51	0.19 (0.095)	0.36	0.39	0.03 (0.015)
208C	0.31	0.19	0.27	0.08 (0.04)	0.20	0.22	0.015 (0.008)
607C	0.35	0.21	0.30	0.09 (0.045)	0.22	0.24	0.015 (0.0075)
C119	0.285	0.15	0.23	0.08 (0.04)	0.17	0.18	0.01 (0.005)
SRD1352/2	0.84	0.29	0.56	**0.27** (0.10)	0.33	0.37	0.05 (0.019)
SRD1352/3	0.57	0.255	0.455	0.20 (0.07)	0.29	0.32	0.04 (0.013)
SRD06038	0.445	0.19	0.35	0.16 (0.065)	0.22	0.25	0.03 (0.012)
SRD06039	0.45	0.21	0.36	0.15 (0.06)	0.23	0.26	0.025 (0.01)
SRD06040	0.35	0.18	0.29	0.11 (0.06)	0.2	0.22	0.02 (0.011)
SRD06041	0.23	0.165	0.215	0.05 (0.03)	0.17	0.185	0.01 (0.006)
ACF CC250	0.315	0.235	0.30	0.065 (0.02)	0.25	0.265	0.015 (0.0045)
FM10/700	0.45	0.22	0.37	0.15 (0.06)	0.24	0.27	0.025 (0.01)
ACF-20	0.78	0.26	0.49	**0.23** (0.025)	0.29	0.325	0.035 (0.004)
C-2132	0.93	0.395	0.67	**0.275** (0.08)	0.44	0.48	0.04 (0.011)
AX-21	0.55	0.44	0.54	0.10 (0.025)	0.46	0.485	0.02 (0.005)
MSC-30	1.06	0.30	0.64	**0.34** (0.09)	0.34	0.395	0.05 (0.013)

**Table 8:** Heat of evaporation Δ*H*_e_ and boundary pressures *P*_1_, *P*_2_, *P*_3_, and *P*_4_ (Figure 1) of various HFCs in the “mild” and “harsh” HeCol cycles.

HFC	Δ*H*_e_^1^, kJ/mol (kJ/g)	*T*_L_/*T*_M_/*T*_H_ = −20/20/35 °C				*T*_L_/*T*_M_/*T*_H_ = −30/3/35 °C			
		Desorption		Adsorption		Desorption		Adsorption	
		*P*_4_, bar	*P*_1_, bar	*P*_2_, bar	*P*_3_, bar	*P*_4_, bar	*P*_1_, bar	*P*_2_, bar	*P*_3_, bar
R32	14.6 (0.28)	9.78	4.05	6.48	14.7	3.23	2.73	7.77	8.91
R134	18.5 (0.18)	3.58	1.33	2.73	5.72	1.09	0.85	2.72	3.21
R152	19.0 (0.29)	3.32	1.25	2.04	5.14	0.97	0.81	2.52	3.03

^1^ at 20 °C.

**Table 9:** Exchanged uptake Δ*w* for various HFCs and adsorbents estimated for the “mild” and “harsh” HeCol cycles.

Cycle Analyzed	*T*_L_/*T*_M_/*T*_H_ = −20/20/35 °C	*T*_L_/*T*_M_/*T*_H_ = −30/3/35 °C
Working Pair Tested	Δ*w, g/g* (g/cm^3^)	Δ*w, g/g* (g/cm^3^)
R32—phenolic rubber [38]	0.90 (0.21)	0.20 (0.046)
R32—208C [41]	0.055 (0.028)	0.013 (0.007)
R32—ACF-20 [23]	0.30 (0.031)	0.06 (0.006)
R32—Maxsorb III [23]	0.53 (0.089)	0.11 (0.019)
R134—SRD 1352/3 [42]	0.13 (0.05)	0.03 (0.011)
R134—GAC [43]	0.24 (0.12)	0.11 (0.06)
R134—ACF-20 [42]	0.28 (0.03)	0.08 (0.008)
R134—Maxsorb III [44]	0.40 (0.068)	0.11 (0.019)
R152—Maxsorb III [38]	0.55 (0.093)	0.17 (0.029)

**Table 10:** Slopes *A* estimated from Equation (3) for various adsorptives involved.

Adsorptive	Methanol	Water	Ammonia	R32
Desorption (4-1)	162	193	100	86
Adsorption (1-3*)	134	170	83	71

**Table 11:** Boundary pressures *P*_1_, *P*_2_, *P*_3_ and *P*_4_ (Figure 1) in the “mild” and “harsh” HeCol cycles for the investigated adsorptives. Pressures are given in mbar for water and methanol and in bar for ammonia and R32.

Adsorptive	*T*_L_/*T*_M_/*T*_H_ = −20/20/35 °C				*T*_L_/*T*_M_/*T*_H_ = −30/3/35 °C			
	Desorption		Adsorption		Desorption		Adsorption	
	*P*_4_	*P*_1_	*P*_2_	*P*_3_	*P*_4_	*P*_1_	*P*_2_	*P*_3_
Water	9.3	1.03	2.89	23.4	0.82	0.38	3.84	7.6
Methanol	54.9	9.95	24.3	123.1	6.71	4.5	32.6	46.7
Ammonia	3.24	1.90	5.27	8.57	1.45	1.19	3.96	4.72
R32	9.78	4.05	6.48	14.7	3.23	2.73	7.77	8.91

## Nomenclature

*A*	slope coefficient, J/(mol K)
*A*_dif_	diffusional flux, Pa m^3^/s
*C*	specific heat capacity, J/(g K)
*D*	average size of grains, mm
*d*_p_	average size of pores, nm
*F*	adsorption potential, J/mol
*H*	enthalpy, J/mol
*M*, *m*	mass, g
*P*	pressure, mbar
*Q*	specific heat, J/g
*S*	surface, m^2^
*S*_sp_	specific surface area, m^2^/g
*T*	temperature, K, °C
*W*	specific power, W/g
*w*	specific adsorbate mass, g/g
wt.%	weight %
*Subscript*	
a	adsorbent
ads	adsorption
Al	aluminum
con	condenser
des	desorption
e	evaporation
ev	evaporator
H	high
in	initial
Kn	Knudsen
L	low
M	medium
m	melting
max	maximal
mol	molecular
p	overall in pores
Pois	Poiseuille
sat	saturation
sen	sensible
us	useful
*Abbreviation*	
AdHEx	adsorbent – heat exchanger
AC	activated carbon
AHT	adsorptive heat transformation
CSPM	composite “salt in porous matrix”
GWP	global warming potential
HeCol	Heat from Cold
HFC	hydrofluorocarbons
R32	difluoromethane
R134	1,1,1,2-tetrafluoroethane
R152	1,1-difluoroethane
